# Disparities in healthcare in psoriatic arthritis: an analysis of 439 patients from 13 countries

**DOI:** 10.1136/rmdopen-2021-002031

**Published:** 2022-05-06

**Authors:** Florian Lucasson, Uta Kiltz, Umut Kalyoncu, Ying Ying Leung, Penélope Palominos, Juan D Cañete, Rossana Scrivo, Andra Balanescu, Emanuelle Dernis, Sandra Meisalu, Adeline Ryussen-Witrand, Martin Soubrier, Sibel Zehra Aydin, Lihi Eder, Inna Gaydukova, Ennio Lubrano, Pascal Richette, Elaine Husni, Laura C Coates, Maarten de Wit, Josef S Smolen, Ana-Maria Orbai, Laure Gossec

**Affiliations:** 1 INSERM, Institut Pierre Louis d'Epidémiologie et de Santé Publique, Sorbonne Université, Paris, France; 2 Herne and Ruhr-Universität, Rheumazentrum Ruhrgebiet, Bochum, Germany; 3 Department of Internal Medicine, Division of Rheumatology, Hacettepe University Faculty of Medicine, Ankara, Turkey; 4 Duke-NUS Medical School, Singapore General Hospital, Singapore; 5 Rheumatology Department, Hospital de Clinicas de Porto Alegre, Porto Alegre, Brazil; 6 Servicio de Reumatología, Hospital Clínic and IDIBAPS, Barcelona, Spain; 7 Rheumatology Unit, Department of Clinical Internal, Anesthesiological and Cardiovascular Sciences, Sapienza Università di Roma, Roma, Italy; 8 Department of Internal Medicine and Rheumatology, “Sf. Maria” Hospital, Carol Davila University of Medicine and Pharmacy, Bucharest, Romania; 9 Rheumatology Unit, Le Mans General Hospital, Le Mans, France; 10 East Tallinn Central Hospital, Tallinn, Estonia; 11 Centre d'Investigation Clinique de Toulouse CIC1436, Inserm, Paul Sabatier University, Toulouse University Hospital, Toulouse, France; 12 Gabriel Montpied Hospital, Clermont-Ferrand, France; 13 The Ottawa Hospital Research Institute, University of Ottawa Faculty of Medicine, Ottawa, Ontario, Canada; 14 Women's College Hospital, University of Toronto, Toronto, Ontario, Canada; 15 North-western State Medical University, St.Petersburg, Russian Federation; 16 Academic Rheumatology Unit, Dipartimento di Medicina e Scienze della Salute “Vincenzo Tiberio", University of Molise, Campobasso, Italy; 17 Service de Rhumatologie, Hopital Lariboisiere Centre Viggo Petersen, Paris, France; 18 Inserm UMR1132 Bioscar, Universite Paris Diderot UFR de Medecine, Paris, France; 19 Department of Rheumatic and Immunologic Diseases, Cleveland Clinic, Cleveland, Ohio, USA; 20 Nuffield Department of Orthopaedics, Rheumatology and Musculoskeletal Sciences, University of Oxford, Oxford, UK; 21 Patient Research Partner, EULAR, Zaltbommel, The Netherlands; 22 Division of Rheumatology, Department of Medicine 3, Medical University of Vienna, Wien, Austria; 23 Division of Rheumatology, Psoriatic Arthritis Program, Johns Hopkins University School of Medicine, Baltimore, Maryland, USA; 24 INSERM, Institut Pierre Louis d'Epidémiologie et de Santé Publique, INSERM, Sorbonne Universite, Paris, France; 25 APHP, Rheumatology Department, Hopital Universitaire Pitie Salpetriere, Paris, France

**Keywords:** Arthritis, Psoriatic, Outcome Assessment, Health Care, Epidemiology

## Abstract

**Objectives:**

Patient care can vary substantially by country. The objective was to explore differences in psoriatic arthritis (PsA) across countries for disease activity, impact and treatments.

**Methods:**

A cross-sectional analysis of 13 countries from the Remission/Flare in PsA study (NCT03119805) of consecutive adult patients with definite PsA was performed. Countries were classified into tertiles by gross domestic product (GDP)/capita. Disease activity (Disease Activity in PsA, DAPSA and Minimal Disease Activity, MDA) and their components, disease impact (patient-reported outcomes) and biological disease-modifying antirheumatic drugs (bDMARDs) were analysed per country and compared between the three tertiles of GDP/capita by parametric and non-parametric tests. We also explored the percentage of patients with significant disease activity (DAPSA >14) and no ongoing bDMARD prescription.

**Results:**

In 439 patients (50.6% male, mean age 52.3 years, mean disease duration 10.1 years), disease activity and disease impact were higher in the lowest GDP/capita countries. DAPSA remission and MDA were attained in the lowest tertile in 7.0% and 18.4% patients, vs 29.1% and 49.5% in the middle tertile and 16.8% and 41.3% in the high tertile, respectively (all p<0.001). bDMARDs use was similar in the tertiles (overall mean 61%). The overall rate of patients with DAPSA >14 and no bDMARDs was 18.5%, and was higher in lower GDP/capita countries (p=0.004).

**Conclusion:**

PsA patients from countries with the lowest GDP/capita, despite similar use of bDMARDs, were more likely to have high disease activity and worse disease impact. There is a need for more equity in healthcare.

**Key messages**

**What is already known about this subject?**

- The country where a patient receives care influences patients’ health status in chronic diseases such as rheumatoid arthritis.
- Country disparities are partly explained by a country’s wealth, which can be measured by gross domestic product (GDP)/capita.

**What does this study add?**

- We explored the role a country’s wealth on outcomes in psoriatic arthritis (PsA), by analysing consecutive patients in an observational study from 13 countries.
- In lower GDP/capita countries, patients with PsA had higher levels of disease activity and more patient-reported impact of disease.
- The use of biological disease-modifying antirheumatic drugs (bDMARDs) was similar across countries. However, in lower GDP/capita countries, more patients in moderate/high disease activity were not receiving a bDMARD.

**How might this impact on clinical practice or further developments?**

- This study highlights differences between countries in the health status of patients with PsA, with more disease activity and less appropriate use of bDMARDs in countries with lower wealth.
- National and international organisations need to promote equity for all patients with PsA. 

## Introduction

Disparities in health are important and pose a challenge for public policies. A country’s wealth is a major factor explaining health disparities. Gross domestic product (GDP) and GDP per capita play a role in the health status of populations.^1^ A striking example is the number of children’s death each year which occur almost only in poor countries: six countries account for 50% of worldwide deaths in children younger than 5 years.^2^ An analysis of the World Bank also showed that, in 2000, a 1% difference in GDP was associated with 12%–14% difference in life expectancy at birth.^3^

Healthcare disparities can be explained by several factors, including access to care, financial restrictions, healthcare provider choices and patient-related factors. Barriers to the implementation of recommended management strategies can come from physicians, linked to impracticality of some of the recommendations but also disagreement with disease activity measures.^4 5^ From the patients’ perspective, fears and beliefs about disease and treatment, which are partly cultural, influence global impact of disease, adherence to therapy and coping patterns.^6–9^

Cardiovascular diseases illustrate inequalities between countries in healthcare. Indeed, while cardiovascular diseases account for 30% of annual global mortality, there are substantive equity gaps, particularly pronounced in low-income countries, in the implementation of cost-effective interventions and provision of quality care, for example, access to stroke unit care which is limited for low-income countries.^10^ When focusing on the field of rheumatology, health disparities may influence two dimensions: disease status (ie, disease activity and impact) and access to treatment. In rheumatoid arthritis (RA), patients from wealthier countries appear to have lower disease activity though results are conflicting for disease impact.^11 12^ For axial and peripheral spondyloarthritis (SpA), disease activity and disease impact appear higher in lower GDP/capita countries.^13^ Regarding access to treatment, in Europe in 2013, among 46 countries, 10 countries did not reimburse biological disease-modifying antirheumatic drugs (bDMARDs) for RA, and patients with RA in lower GDP/capita countries had less access to bDMARDs.^14^ Similarly, in SpA a lower use of bDMARDs and a higher use of conventional synthetic DMARDs (csDMARDs) was observed in countries with lower GDP in 2015.^15^

Psoriatic arthritis (PsA) is a complex inflammatory disease that presents a wide spectrum of clinical patterns^16^ and differing management recommendations, which may lead to country disparities.^17 18^ In PsA, given the lack of large cohorts or real-world datasets, health disparities across countries have been little explored.^19–21^

To assess health disparities in PsA, it is of interest to explore disease activity, in particular using the Disease Activity in PsA (DAPSA) score or Minimal Disease Activity (MDA) binary classification,^22 23^ disease impact, through patient-reported outcomes (PROs), treatment use and the proportion of patients with moderate to high disease activity and no bDMARDs, which may reflect suboptimal management.

The objective of this analysis was to explore the differences between countries in PsA outcomes and treatment choices, and the role of GDP/capita in these differences.

## Methods

### Study population and study design

The Remission/Flare in PsA (ReFlaP) study was a prospective, multicentre international, observational study, as reported elsewhere.^24–27^ Briefly, the study took place in 21 centres in 14 countries, including 8 countries across Europe (Austria, Estonia, France, Germany, Italy, Romania, Spain and the UK), Brazil, Canada, the Russian Federation, Singapore, Turkey and the USA between June 2017 and August 2018 (NCT03119805). In this analysis, one country (Austria) was excluded due to insufficient recruitment (seven patients). Consecutive adult patients with a diagnosis of PsA as defined by their rheumatologist and more than 2 years of disease duration were recruited. Investigators were advised to consider the CASPAR criteria for classification of PsA. Overall, 97.5% of the patients included met the CASPAR criteria.

### Data collection

#### GDP/capita

Countries were ordered by GDP/capita, according to the International Monetary Fund 2017 database.^28^ Countries were analysed separately, and classified into tertiles by GDP/capita.

#### Disease activity and disease impact

PsA disease activity was assessed using DAPSA (continuous score) and MDA (yes/no).^17 22 23^ DAPSA is calculated as the sum of 66 swollen joint count (SJC66) and 68 tender joint count (TJC68), patient-reported pain, patient global assessment (PGA) and C reactive protein (CRP, mg/dL). A DAPSA score less than 4 represents remission, and a score less than 14 represents low disease activity (LDA).^22^ MDA includes seven PsA disease activity criteria; if five out of seven are met, the patient is considered in MDA. TJC and SJC, dactylitis, tender entheseal points (Leeds Enthesitis Index),^29^ body surface area of psoriasis, CRP and physician global assessment of PsA were also analysed separately.

Disease impact was assessed through the PsA Impact of Disease questionnaire, PsAID12, which comprises 12 questions on the impact of PsA (range, 0–10 where higher numbers indicate worse status), Health Assessment Questionnaire (HAQ) and PGA.^30–33^

#### Treatments

Current csDMARD intake was collected (methotrexate, leflunomide, sulfasalazine and other csDMARDs). Current bDMARDs were also collected (TNF alpha inhibitors, ustekinumab, secukinumab and other bDMARDs) as well as JAK inhibitors (as free text) and current oral glucocorticoids.

In order to better analyse inequity in the management of PsA, we explored patients with significant disease activity (ie, moderate to severe disease activity) according to DAPSA (DAPSA>14) and as sensitivity analysis, patients not in MDA, who did not receive bDMARDs at the time of the visit. We considered these exploratory endpoints as a potential surrogate of suboptimal management. The decision to intensify the treatment at the visit due to disease activity was also collected.

#### Other data collected

General characteristics collected included demographic characteristics such as age, sex, years of studies and disease characteristics such as disease duration and predominant type of PsA (axial or peripheral). A validated comorbidity index was also collected (Groll Functional Comorbidity Index).^34^

### Statistical analysis

For this post hoc exploratory analysis, ReFlaP patients were analysed per country and compared between the three tertiles of GDP/capita by parametric and non-parametric tests. Exploratory analyses were performed on men and women separately. Quantitative variables were expressed as mean and SD, median and IQR and were compared by Kruskall-Wallis test or Mann-Whitney-Wilcoxon test, as appropriate. Weighted means per tertile (ie, accounting for varying sample sizes per country) were computed. Qualitative variables were expressed as numbers and percentages and were compared by χ^2^ test. We did not adjust for covariables within a country given the limited number of patients in some countries. Cohen’s effect size was calculated for quantitative variables assessing disease activity and impact, comparing the lowest GDP/capita tertile to the two other tertiles.^35^

There was no imputation of missing data. Patients with missing data for DAPSA, MDA or physician global assessment were excluded from the disease activity analysis. Patients with bDMARDs missing data were excluded from the treatment analysis. The main analyses for disease activity were repeated without the outlier country (the Russian Federation). An a posteriori sample size calculation indicated that to demonstrate a difference in remission/LDA rates (based on MDA) of 25% between the lower tertile of GDP/capita and the 2 higher tertiles of GDP/capita, 123 patients are needed (41 in the lower tertile and 82 in the 2 higher tertiles, enrollement ratio of 2:1). This calculation was made using clincalc.com based on an expected rate of remission/LDA of 45% in the lower tertile of GDP/capita and 20% in the two higher tertiles, with an alpha level of 0.05 and a power of 0.80.^24–27^

## Results

Among the 459 patients included in 13 countries, 439 had disease activity data available and 410 had treatment data available. In the disease activity population, there were 218 men (50.6%), mean age was 52.3 (SD 12.6) years and mean disease duration was 10.1 (SD 8.1) years. The treatment population was globally similar to the disease activity population (data not shown). Countries were ordered by GDP/capita and classified into tertiles (lowest tertile: Brazil, Turkey, Russian Federation, Romania and Estonia; middle tertile: Spain, Italy, UK and France; highest tertile: Canada, Germany, USA and Singapore). In the tertile of countries with the lowest GDP/capita, there were fewer men (36.8%), patients were slightly younger (mean age 49.6 (SD 12.3) years) than in the other tertiles; disease duration was longer than in countries with high GDP/capita but shorter than in countries with middle GDP/capita (table 1).

**Table 1:** Characteristics, disease activity and disease impact of 439 PSA patients from 13 countries grouped by GDP/capita

	Patients in lowest country GDP/capita tertile (N=114)	Patients in middle country GDP/capita tertile (N=182)	Patients in highest country GDP/capita tertile (N=143)	P value between the tertiles (Effect size)* (95% CI)
Patients characteristics				
Age (years), mean (SD)	49.6 (12.3)	54 (12.4)	52.2 (12.7)	
Gender male, n (%)	42 (36.8)	105 (60.0)	71 (50.0)	
Disease duration (years), mean (SD)	9.6 (7.0)	11.9 (8.6)	8.1 (7.6)	
Years of studies, mean (SD)	11.0 (3.6)	12.8 (3.1)	14.4 (3.2)	
Groll comorbidity index, mean (SD)	1.5 (2.3)	0.6 (0.9)	1.2 (1.4)	
Predominant peripheral involvement, n (%)	95 (88.8)	148 (88.1)	130 (95.6)	
Predominant axial involvement, n (%)	12 (11.2)	15 (8.9)	5 (3.7)	
Disease activity				
MDA state attained, n (%)	21 (18.4)	90 (49.5)	59 (41.3)	<0.001
DAPSA remission, n (%)	8 (7.0)	53 (29.1)	24 (16.8)	<0.001
DAPSA, mean (SD)	21.2 (20.4)	13.6 (15.5)	15.8 (14.5)	<0.001 (0.40 (0.19 to 0.62))
Swollen joint count (0–66), mean (SD)	3.8 (11.1)	1.2 (5.4)	2.1 (3.7)	<0.001 (0.31 (0.10 to 0.53))
Tender joint count (0–68), mean (SD)	5.7 (10.3)	3.3 (7.8)	4.9 (9.1)	0.002 (0.19 (−0.02 to 0.40))
Current dactylitis, n (%)	8 (7.1)	8 (4.5)	11 (7.9)	0.416
Leeds Enthesitis Index, mean (SD)	0.6 (1.4)	0.5 (1.2)	0.6 (1.4)	0.325 (0.08 (−0.14 to 0.29))
Body surface area of psoriasis >5%, n (%)	20 (17.5)	14 (7.7)	8 (5.6)	0.003 (0.39 (0.17 to 0.60))
Elevated C reactive protein (>5 mg/L), n (%)	10 (8.8)	15 (8.2)	2 (1.4)	0.016 (0.06 (−0.16 to 0.27))
Physician global assessment of PsA (0–10), mean (SD)	4.1 (2.5)	2.6 (2.5)	2.9 (2.4)	<0.001 (0.58 (0.36 to 0.79))
Disease impact				
PsAID12 (0–10), mean (SD)	4.3 (2.4)	2.9 (2.3)	3.3 (2.5)	<0.001 (0.50 (0.28 to 0.72))
Patient global assessment of PsA (0–10), mean (SD)	5.1 (2.6)	3.5 (2.8)	4.0 (3.0)	<0.001 (0.49 (0.28 to 0.71))
HAQ (0–3), mean (SD)	0.9 (0.7)	0.5 (0.6)	0.7 (0.7)	<0.001 (0.48 (0.26 to 0.69))

Lowest country GDP/capita tertile: Brazil, Turkey, Russian Federation, Romania, Estonia; middle tertile: Spain, Italy, UK, France; highest tertile: Canada, Germany, USA and Singapore.

Data were missing for predominant type of PsA (n=28), years of studies (n=23), disease duration (n=14), age (n=12), gender (n=8), current dactylitis (n=8) and PsAID12 (n=1). Percentage of male and predominant type of PsA are percentages of available data.

*Effect size (95% CI) comparing the lowest GDP/capita tertile to the two others tertiles (see also online supplemental table 1).

DAPSA, Disease Activity in PSoriatic Arthritis; GDP, gross domestic product; HAQ, Health Assessment Questionnaire; MDA, Minimal Disease Activity; PsA, Psoriatic Arthritis; PsAID, PsA Impact of Disease questionnaire.

10.1136/rmdopen-2021-002031.supp1 Supplementary data

### Disease activity

**Figure 1:**
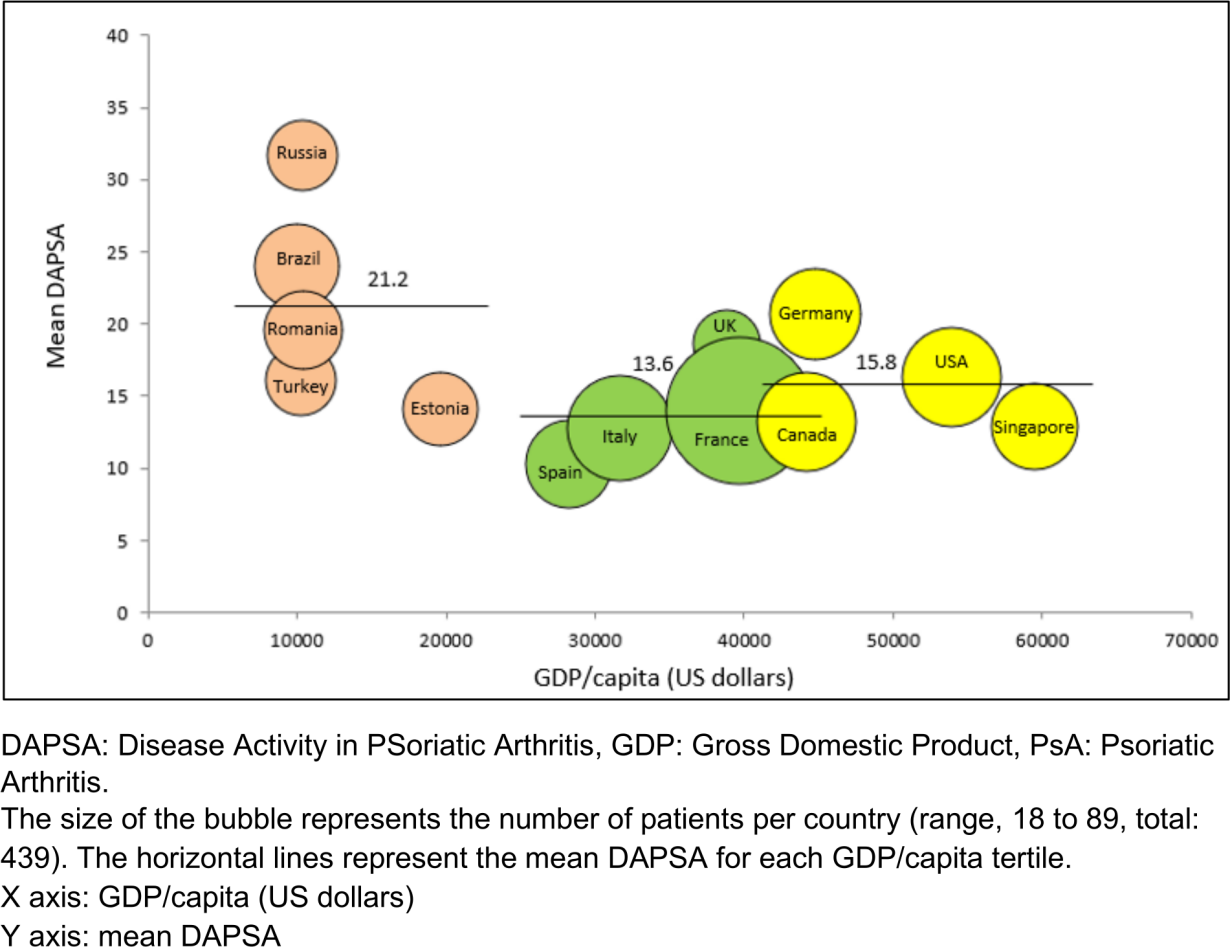
Mean DAPSA mapped against country GDP/capita for PSA patients from 13 countries.

Disease activity was highest in the lowest GDP/capita countries (table 1 and figure 1). DAPSA remission was achieved by only 7.0% of patients in the tertile of countries with low GDP/capita, versus 29.1% and 16.8% in the middle and high GDP/capita tertile respectively (p<0.001). The range of DAPSA remission was 2.9% (Germany) to 43.3% (Spain) (data not shown). Mean DAPSA was highest in the Russian Federation and lowest in Spain (figure 1 and online supplemental table 1).

Similar results were observed with MDA, attained in 18.4%, 49.5% and 41.3% of patients in the lowest to highest GDP/capita tertiles, respectively (p<0.001). A sensitivity analysis was performed excluding the Russian Federation (which was an outlier) from the lowest tertile of GDP/capita and the global result remained unchanged: mean DAPSA was higher in the lowest GDP/capita tertile (p<0.001). Another sensitivity analysis explored if the link between skin psoriasis and DAPSA was similar in the tertiles of countries, and this was confirmed (data not shown).

Components of disease activity were worse in the lowest tertile of GDP/capita, in particular for SJC and body surface area of psoriasis (table 1). The only exceptions were dactylitis and the Leeds Enthesitis Index which were similar across all tertiles. Effect sizes comparing the lowest tertile of GDP/capita to the other tertiles ranged 0.19–0.40 for joint counts, skin and DAPSA (table 1 and online supplemental table 2). The effect size was non-significant for enthesitis and CRP (table 1 and online supplemental table 2). While there were some differences between men and women, they did not affect the overall association with country GDP/capita (data not shown).

10.1136/rmdopen-2021-002031.supp2 Supplementary data

#### Disease impact

Disease impact was worst in the lowest GDP/capita countries (table 1). In these countries, PsAID12 score was ≤4.0 (threshold of the patient acceptable symptom state) in 64.0%, 80.8% and 74.6% of patients in the lowest to highest GDP/capita tertiles, respectively. PGA was also higher in the lowest GDP/capita tertile (table 1). The impact in terms of functional capacity was more significant in the lowest tertile of GDP/capita (table 1). The lowest GDP/capita tertile had a small to moderate effect size on PROs with an effect size ranging 0.48–0.50 (table 1 and online supplemental table 2).

#### Treatment

There was a slightly lower prescription of bDMARDs in the lowest tertile of GDP/capita, not reaching statistical significance, with an overall mean of 61.0% (table 2). Regarding csDMARDs, methotrexate use was higher in the lowest GDP/capita tertile: 63.3% in the lowest GDP/capita tertile, vs 46.5% in the middle tertile and 52% in the highest tertile (p=0.035) (table 2). Use of oral glucocorticoids was higher in the lowest and the highest GDP/capita tertiles reaching almost 26% of patients (table 2).

**Table 2:** Treatment use in 13 countries in 410 patients, grouped by GDP/capita

	Overall (N=410)	Patients in lowest country GDP/capita tertile (N=101)	Patients in middle country GDP/capita tertile (N=180)	Patients in highest country GDP/capita tertile (N=129)	P value between the tertiles
bDMARDs use, n (%)	250 (61.0)	54 (53.5)	113 (62.8)	83 (64.3)	0.197
csDMARDs use, n (%)	237 (61.4)	67 (72.8)	93 (54.7)	77 (62.1)	0.016
Methotrexate use, n (%)	200 (52.2)	57 (63.3)	79 (46.5)	64 (52.0)	0.035
Oral glucocorticoids use, n (%)	64 (17.5)	21 (25.6)	19 (11.6)	24 (20.0)	0.016
High or moderate disease activity (DAPSA >14) and no bDMARD use, n (%)	76 (18.5)	30 (29.7)	27 (15.0)	19 (14.7)	0.004*

Lowest country GDP/capita tertile: Brazil, Turkey, Russian Federation, Romania, Estonia; middle tertile: Spain, Italy, UK, France; highest tertile: Canada, Germany, USA and Singapore.

Data were missing for glucocorticoids (n=44), csDMARDs (n=24) and methotrexate (n=27). Percentages are percentages of available data.

*Specific comparisons were made between tertiles: lowest vs middle (p=0.009), lowest vs highest (p=0.007), middle vs highest (p=0.882).

bDMARDs, biological disease-modifying antirheumatic drugs; csDMARDs, conventional synthetic DMARDs; DAPSA, Disease Activity in PSoriatic Arthritis; GDP, gross domestic product.

**Figure 2:**
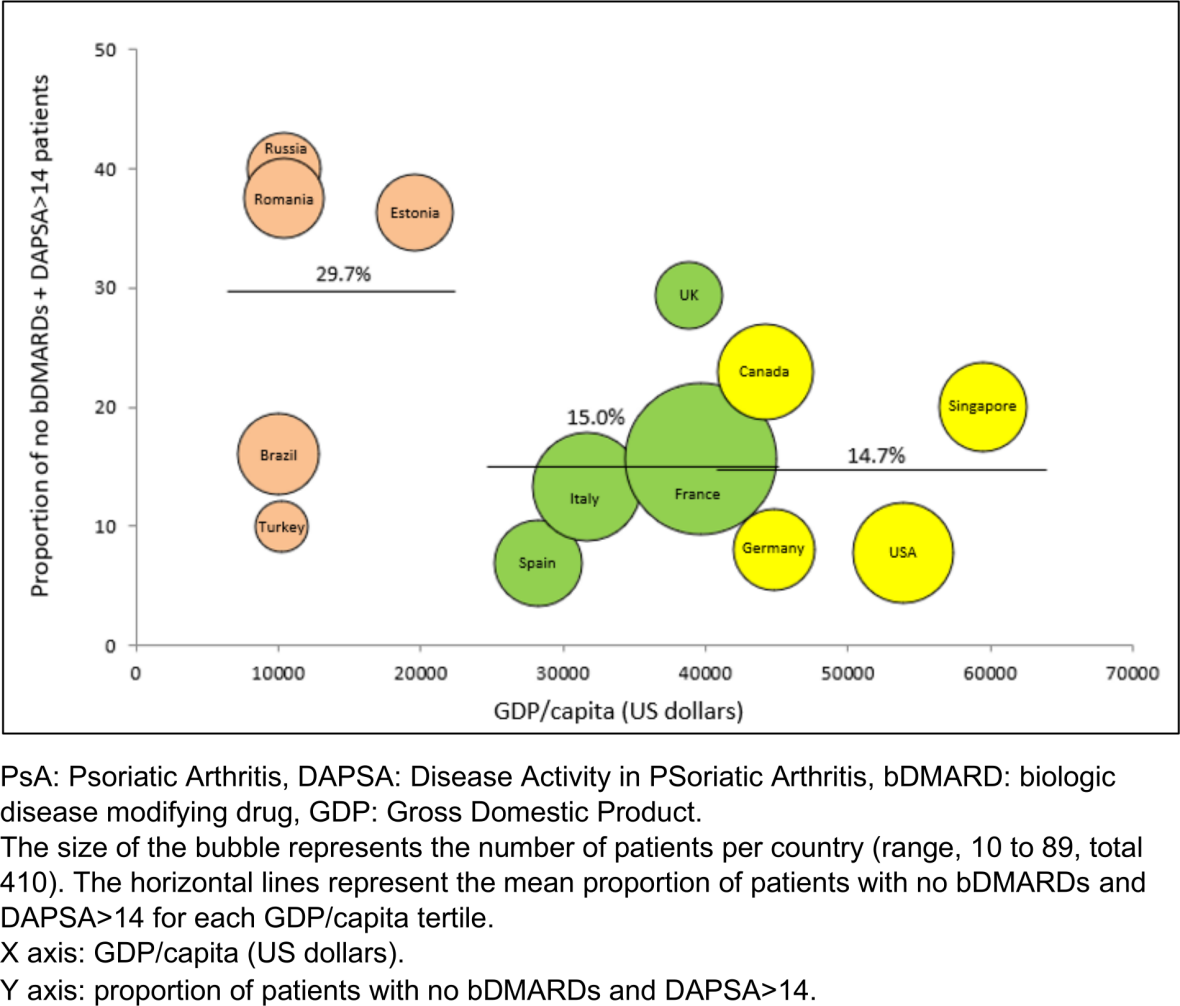
Proportion of PSA patients with moderate to high disease activity (DAPSA >14) not receiving a bDMARD in 13 countries, ordered by increasing GDP/capita.

The overall rate of no bDMARDs use in patients with moderate/high disease activity (DAPSA >14) was 18.5% (76/410) (table 2). This rate ranged from 6.9% (Spain) to 40.0% (Russian Federation) (figure 2). A link was seen with the country and the tertiles of countries according to GDP/capita, with a rate close to 30.0% of no bDMARDs+DAPSA>14 patients in the lowest GDP/capita tertile (table 2 and figure 2). Similar results were seen for patients not in MDA and no bDMARDs. The overall rate of no bDMARDs use in patients not in MDA was 25.9% (106/410) with percentages ranging from 17.8% in the highest tertile to 41.6% in the lowest tertile of GDP/capita (p<0.001).

Of note, 40 of these 76 patients (52.6%) were proposed treatment intensification during the visit. These treatment changes were mainly performed in wealthier countries: 20.0% (6/30), 66.7% (18/27) and 84.2% (16/19) respectively in the three tertiles.

Among no bDMARDs +DAPSA>14 patients, functional capacity was lower than in the other patients, as expected (mean HAQ-DI 1.0 (SD 0.6) vs 0.6 (SD 0.6), respectively, p<0.001).

## Discussion

In this study exploring differences of disease patterns and treatment choices in PsA between 13 countries, we observed that in the lowest GDP/capita countries, patients had higher disease activity levels, measured by DAPSA and MDA, but also by components of disease activity, in particular SJC and body surface area of psoriasis, as well as higher patient-reported disease impact. In addition, outcomes regarding disease activity and impact were not better in the highest tertile compared with the middle tertile of GDP/capita. Furthermore, PsA patients with high or moderate disease activity appeared less likely to be treated with bDMARDs in low GDP/capita countries.

This analysis has strengths and limitations. Given the low number of patients per country, our study should be seen as an eye-opening first look into the disparities between countries in PsA, which needs to be confirmed by larger studies. However, the present results are useful to raise awareness of health disparities in PsA. Recruitment occurred in tertiary care centres, leading to a high rate of patients receiving bDMARDs, which may limit external validity. Nevertheless, the international large-scale recruitment of consecutive patients with PsA improves generalisability. The number of patients in each country was limited and some countries were over-represented; however, frequencies of DAPSA remission and MDA were globally similar to other studies, which supports the validity of the present findings.^36 37^ Despite the fact that countries from Europe and North America were over-represented in this analysis, our sample of countries had a wide range of GDP/capita. Due to the low number of patients per country, we considered the fact that outlier countries may have biased our results. However, a sensitivity analysis excluding the Russian Federation confirmed our findings. The cross-sectional nature of our data allows a snapshot of PsA management. In PsA studies, given the lack of consensus, it is difficult to choose a definition of remission; the scores used in this analysis, DAPSA and MDA, are recommended by the international T2T taskforce.^17 38^ Patients with moderate to high disease activity and no bDMARDs were analysed as a potential surrogate of suboptimal management. However, the absence of prescription of bDMARDs can be explained by elements other than disease activity, such as the existence of contraindications, patient choice or a too high out-of-pocket cost for the patient. Some of these elements are difficult to assess and they were not collected in this study.^17 39^ Imputation of missing data was not performed (online supplemental table 3), which can be discussed as a weakness, since methods to impute missing data can be applied and may in some cases modify the results.

10.1136/rmdopen-2021-002031.supp3 Supplementary data

We evidenced higher disease activity in lower GDP/capita countries. This finding in PsA is consistent with previous studies in different rheumatic diseases. Indeed, in the large-scale QUEST-RA (Quantitative Standard Monitoring of Patients with Rheumatoid Arthritis) and ASAS-COMOSPA (Assessment in SpondyloArthritis Inter-national Society-Comorbidities in SpondyloArthritis) studies (6004 RA patients and 3370 SpA patients, respectively), patients in low GDP countries had higher disease activity levels.^11 12^ In PsA, few studies have been conducted on this subject. A survey compared differences between patients in Spain and in other European countries, but only for skin psoriasis.^20^ In a multinational study of 3714 patients across 18 countries, patients failing to respond to immunomodulatory treatment had worse disease severity according to physician global assessment, in Turkey and Middle Eastern countries.^21^ These studies did not explore the full spectrum of the disease across countries. In our analysis, patient-perceived impact, physician global assessment, SJC and skin disease were higher in lower GDP/capita countries whereas enthesitis and CRP were not, with the highest differences observed for PROs and physician global assessment, as evidenced by effect sizes. Our initial hypothesis was that disease activity would be higher in lower GDP/capita countries whereas disease impact might be higher in the richer countries, since expectations differ across cultures and countries.^13^ However, this was not confirmed. This indicates a coherence between activity and impact in PsA. Enthesitis is a complex manifestation to assess,^40^ and was not different across countries.

It is noteworthy that outcomes regarding disease activity as well as disease impact were not better, and perhaps even slightly worse, for patients living in the highest tertile compared with the middle tertile of GDP/capita. If this finding is confirmed in wider studies, an explanation for this result could be that care in tertiary centres from the middle GDP/capita tertile may be similar to the highest GDP/capita tertile. Another explanation could be that there is variability between countries in health coverage and reimbursement rules, with high out-of-pocket expenses in some of the highest tertile countries, leading to a lack of care for some patients.

Our main finding is that a patient’s country plays a role in their PsA outcomes. In this analysis we explored the role of the country’s wealth represented by GDP/capita. Indeed, a country’s GDP and GDP/capita have a major influence on health although the link is complex.^41 42^ We are well aware that GDP/capita is not the only socioeconomic indicator for comparing countries in the healthcare domain. Indeed, healthcare systems can be very different between countries and other indicators exist such as current expenditure on health as a percentage of GDP, private household out-of-pocket expenditure, or indicators of access to care such as the number of health professionals per 1000 inhabitants or total number of hospital beds per 10 000 inhabitants.^43 44^ It is difficult to determine causality for the higher disease activity observed in lower GDP/capita countries. Indeed, composite scores were higher in these countries, but our analyses do not allow to establish if joints, skin or other elements were the driving factors.^45^ Limited access to some treatments in some GDP/capita countries has been well documented.^14 15^ It is possible that biosimilars may increase access to treatments in less wealthy countries; this study did not explore this aspect.

In our analysis, there was not a linear relationship between GDP and access to treatment. Access to treatment is determined by several factors, including availability, pricing/funding and acceptability.^46^ Availability is affected by market size and health policies related to low income and a low percentage of the GDP allocated to health budgets. Thus, even in some middle or high GDP/capita countries, the number of biological options can be limited. Pricing/funding is particularly important in low-income countries; in 2007, for RA, the average price of bDMARDs as well as the average expenditure per patient were negatively associated with GDP/capita.^47^ Acceptability is a more complex concept which involves physician and patient barriers.^24^

Regarding treatment use, we found that bDMARDs were prescribed similarly in all GDP/capita tertiles whereas methotrexate was more frequent in the lower GDP/capita countries. However, disease activity and impact were worse in these lower GDP/capita countries despite a greater prescription of treatments. This may indicate that physicians may not always prescribe treatment in line with current recommendations. In such a case, patients who should benefit from bDMARDs may be receiving csDMARDs instead. The rate of patients with significant disease activity and no bDMARDs was more important in lower GDP/capita countries, which supports this hypothesis. Furthermore, although many of these patients with active disease received treatment intensification during the visit, these treatment changes were also less frequent in lower GDP/capita countries. In the analysis of access to bDMARDs in patients with significant disease activity, there appears to be a divide in the lowest GDP/capita tertile between Russian Federation/Romania/Estonia on the one hand and Turkey/Brazil on the other hand. GDP/capita may not explain all the differences observed and Russian Federation/Romania/Estonia share cultural factors, as Eastern European countries, and share some reimbursement rules for bDMARDs. However, we are unable to confirm these hypotheses with the data available to us; further studies are needed.

Of note, we did not collect treatment dose which could be a factor explaining outcomes’ differences between countries. Indeed, physicians may prescribe methotrexate at lower, and therefore, less effective, doses in some countries, due to fear of adverse effects for instance.^48^ Actual intake of treatment (adherence) was also not collected.

Country differences may be linked to many other elements than GDP. In this study, we collected the information on reimbursement patterns for drugs in the centres involved in ReFlaP; however, the data could not be analysed since reimbursement was extremely variable.

Patient factors may play a role in countries differences. Given the heterogeneity of PsA, genetic factors and disease presentation vary across countries and may influence outcomes.^49^ Patients’ lifestyle, as well as personal socioeconomic and educational status, could influence quality of care since a social gradient in health runs from top to bottom of the socioeconomic range (the lower an individual’s socioeconomic position the worse their health) and affects all countries.^1 3 50^ In our analysis, patients from the first tertile of GDP/capita had a lower level of education, which may partly explain the differences observed between countries. Quality of care is also affected by patient barriers regarding PsA management which stem from fears and beliefs about drugs or disease and are partly cultural.^7^ Finally, culture and environment may also drive differences across countries.

In conclusion, differences were found across countries and may be related to healthcare systems (affordability and availability of healthcare), patient factors (acceptability of care, lifestyle) and physician factors (compliance with recommendations). These country differences should be confirmed by wide-scale systematic studies. The descriptive analyses performed here should also be supplemented with causality analyses in the future. If confirmed, directions for the future include political measures to generalise access to drugs, patient education programmes and dissemination of management recommendations. Such projects would promote more equity in healthcare in rheumatology.

## Data Availability

Data are available on reasonable request. The ReFlaP data are available from the last author on reasonable request.
