# Correction: Disparities in health care in psoriatic arthritis: an analysis of 439 patients from 13 countries

**DOI:** 10.1136/rmdopen-2021-002031corr1

**Published:** 2022-05-26

Lucasson F, Kiltz U, Kalyoncu U, *et al*. Disparities in healthcare in psoriatic arthritis: an analysis of 439 patients from 13 countries. *RMD Open* 2022;8:e002031.

Juan Canete has been updated to Juan D Cañete.

