# Supplementary material for: Disparities in healthcare in psoriatic arthritis: an analysis of 439 patients from 13 countries
Source: RMD Open. 2022 May 6;8(1):e002031. doi: 10.1136/rmdopen-2021-002031 (PMC9083399; doi:10.1136/rmdopen-2021-002031)
Supplement: Supplementary data [file rmdopen-2021-002031supp001.pdf]

Supplementary Table 1. Mean DAPSA for PsA patients according to country

Tertile	Lowest country GDP/capita					
Country	Brazil (N=29)	Russian Federation (N=20)	Romania (N=24)	Turkey (N=19)	Estonia (N=22)	Overall (N=114)
DAPSA, mean (SD)	24.0 (29.4)	31.7 (23.4)	19.6 (15.9)	16.1 (8.8)	14.1 (7.8)	21.2 (20.4)

Tertile	Middle country GDP/capita				
Country	Spain (N=30)	Italy (N=45)	UK (N=18)	France (N=89)	Overall (N=182)
DAPSA, mean (SD)	10.3 (14.2)	12.8 (11.4)	18.6 (20.8)	14.0 (16.5)	13.6 (15.5)

Tertile	Highest country GDP/ capita				
Country	Germany (N=34)	Canada (N=39)	Singapore (N=30)	USA (N=40)	Overall (N=143)
DAPSA, mean (SD)	20.7 (18.4)	13.2 (11.6)	12.9 (11.9)	16.3 (14.4)	15.8 (14.5)

DAPSA: Disease Activity in PSoriatic Arthritis, PsA: Psoriatic Arthritis, GDP: Gross Domestic Product
