# Supplementary material for: Disparities in healthcare in psoriatic arthritis: an analysis of 439 patients from 13 countries
Source: RMD Open. 2022 May 6;8(1):e002031. doi: 10.1136/rmdopen-2021-002031 (PMC9083399; doi:10.1136/rmdopen-2021-002031)
Supplement: Supplementary data [file rmdopen-2021-002031supp002.pdf]

Supplementary Table 2. Calculation of effect size comparing the lowest GDP/capita tertile to the two other tertiles

	Patients in lowest country GDP/capita tertile (N=114)	Patients in middle and high country GDP/capita tertiles (N=325)	Effect size [95% CI]
DAPSA, mean (SD)	21.2 (20.4)	14.5 (15.1)	0.40 [0.19;0.62]
Swollen joint count (0-66), mean (SD)	3.8 (11.1)	1.6 (4.8)	0.31 [0.10;0.53]
Tender joint count (0-68), mean (SD)	5.7 (10.3)	4.0 (8.4)	0.19 [-0.02;0.40]
CRP (mg/dL), mean (SD)	1.8 (4.6)	1.5 (5.6)	0.06 [-0.16;0.27]
Leeds enthesitis index, mean (SD)	0.6 (1.4)	0.5 (1.3)	0.08 [-0.14;0.29]
Body surface area of psoriasis (analysed as a continuous variable), mean (SD)	4.5 (6.8)	2.6 (4.0)	0.39 [0.17;0.60]
Physician global assessment of PsA (0-10, mean (SD)	4.1 (2.5)	2.7 (2.4)	0.58 [0.36;0.79]
PsAID12, mean (SD)	4.3 (2.4)	3.1 (2.4)	0.50 [0.28;0.72]
Patient global assessment of PsA (0-10), mean (SD)	5.1 (2.6)	3.7 (2.9)	0.49 [0.28;0.71]
HAQ (0-3), mean (SD)	0.9 (0.7)	0.6 (0.6)	0.48 [0.26;0.69]

PsA: Psoriatic Arthritis, GDP: Gross Domestic Product, MDA: Minimal Disease Activity, DAPSA: Disease Activity in PSoriatic Arthritis, PsAID: PsA Impact of Disease questionnaire, HAQ: Health Assessment Questionnaire.
Lowest country GDP/capita tertile: Brazil, Turkey, Russian Federation, Romania, Estonia; middle tertile: Spain, Italy, UK, France; highest tertile: Canada, Germany, USA and Singapore.
95% CI: 95% Confidence Interval
