# Supplementary material for: Disparities in healthcare in psoriatic arthritis: an analysis of 439 patients from 13 countries
Source: RMD Open. 2022 May 6;8(1):e002031. doi: 10.1136/rmdopen-2021-002031 (PMC9083399; doi:10.1136/rmdopen-2021-002031)
Supplement: Supplementary data [file rmdopen-2021-002031supp003.pdf]

Supplementary Table 3. Missing data for key outcomes in each tertile of GDP/capita

A) In 439 patients (disease activity population)

	Patients in lowest country GDP/capita tertile (N=114)	Patients in middle country GDP/capita tertile (N=182)	Patients in highest country GDP/capita tertile (N=143)
MDA state attained, n (%)	0	0	0
DAPSA remission, n (%)	0	0	0
DAPSA, n (%)	0	0	0
Swollen joint count (0-66), n (%)	0	0	0
Tender joint count (0-68), n (%)	0	0	0
Current dactylitis, n (%)	1 (0.9)	3 (1.6)	4 (2.8)
Leeds enthesitis index, n (%)	0	0	0
Body surface area of psoriasis ≥5%, n (%)	0	0	0
Elevated C-reactive protein (CRP>5mg/L), n (%)	0	0	0
Physician global assessment of PsA (0-10), n (%)	0	0	0
PsAID12 (0-10), n (%)	0	0	1 (0.9)
Patient global assessment of PsA (0-10), n (%)	0	0	0
HAQ (0-3), n (%)	0	0	0

B) In 410 patients (treatment population)

	Patients in lowest country GDP/capita tertile (N=101)	Patients in middle country GDP/capita tertile (N=180)	Patients in highest country GDP/capita tertile (N=129)
bDMARDs use, n (%)	0	0	0
csDMARDs use, n (%)	9 (8.9)	10 (5.6)	5 (3.9)
Methotrexate use, n (%)	11 (10.9)	10 (5.6)	6 (4.7)
Oral glucocorticoids use, n (%)	19 (18.8)	16 (8.9)	9 (7.0)
